# Effects of Different Proteases on Protein Digestion In Vitro and In Vivo and Growth Performance of Broilers Fed Corn–Soybean Meal Diets

**DOI:** 10.3390/ani13111746

**Published:** 2023-05-25

**Authors:** Mengli Zheng, Yan Bai, Yingxia Sun, Jing An, Qinghua Chen, Tieying Zhang

**Affiliations:** 1 State Key Laboratory of Animal Nutrition, Institute of Animal Science, Chinese Academy of Agricultural Sciences, Beijing 100193, China; 2 College of Animal Science and Technology, Hunan Agricultural University, Changsha 410128, China; chqh314@163.com; 3 College of Animal Science, Shanxi Agricultural University, Jinzhong 030801, China

**Keywords:** protease, broiler, in vitro, in vivo, protein digestibility, pH

## Abstract

**Simple Summary:**

With the increase of protease types and products, it is time-consuming and laborious to evaluate the effect of protease on feed protein utilization with animal experiments, and it is not conducive to evaluate a large number of samples in a short time. The purpose of this study was to quickly evaluate the effects of four proteases (acidic, neutral, alkaline and keratinase) on feed ingredients (corn gluten meal, corn and soybean meal) using an in vitro method to determine the optimal dosage of each protease for corn gluten meal and corn and soybean meal, and to explore the factors affecting the effect of proteases. In addition, this research also carried out animal experiments to verify the effect of protease on the corn–soybean meal diet of 31-day-old broilers.

**Abstract:**

This study was conducted to investigate the effects of different proteases alone or in combination on protein digestibility of broilers. In vitro, the properties of four proteases in broilers, including acidic protease (AcP), alkaline protease (AlP), neutral protease (NeP) and keratinase (Ker), on endogenous protease activity and their effects on protein digestibility of common ingredients in broiler diets were investigated using a gut-mimicking model. In vivo, 640 1-day-old male broilers were randomly divided into 8 groups of 10 with 8 replicates of 10 birds per replicate cage. Eight dietary treatments included a corn–soybean meal basal diet (control), and the basal diet with 1.6 U AcP/g, 0.8 U NeP/g, 0.8 U AlP/g, 0.4 U Ker/g, 1.6 U AcP/g + 0.8 U NeP/g, 1.6 U AcP/g + 0.8 U AlP/g, or 1.6 U AcP/g + 0.4 U Ker/g added. The experiment lasted for 31 days. The results showed that the optimum pH values of AcP, NeP, AlP and Ker were 3.0, 9.0, 11.0 and 11.0 in vitro, respectively. Ker recovery proportion was 37.68% at pH 3.3–6.2. AcP alone or in combination with NeP, AlP or Ker increased in vitro crude protein digestibility (IVCPD) and decreased ileal apparent digestibility of crude protein in 31-day-old broilers (*p* < 0.05). All protease supplementation reduced the ileal apparent digestibility of amino acids compared to the control (*p* < 0.05). Acidic protease had a positive effect on trypsin and chymotrypsin activities, while AlP and Ker showed a negative effect. In vivo, average daily gain and average daily feed intake were significantly (*p* < 0.05) increased in broiler diets supplemented with AcP compared to the control group. When adding exogenous proteases to broiler diets, their sensitivity to digestive pH and their negative effects on endogenous protease activity, dosage and combination effects should be taken into account. In addition, the properties and dosage of proteases and the protein level in the feed should be considered.

## 1. Introduction

The digestion and utilization of dietary nutrients by broilers can be fully considered in combination with their physiological structure, gastrointestinal digestion and absorption characteristics and dietary structure characteristics [1,2,3]. After digestion of feed nutrients in the crop and stomach of broilers, the chyme is transferred into the small intestine to continue digestion. In this process, the endogenous protease secreted by the body plays an important role in the degradation of protein macromolecules.

Studies on the digestibility of crude protein (CP) and amino acid (AA) in broilers showed that valuable proteins passed through the gastro-intestinal tract (GIT) without complete digestion [4,5]. Soybean meal (SBM), as one of the most commonly used protein sources for broilers globally, has the advantages of a higher digestibility and good amino acid balance [6]. However, soybean protein is rich in a variety of anti-nutritional factors, such as soybean protease inhibitors, particularly the Kunitz trypsin inhibitor (KTI) of soybean, which is a β-sheet protein with abnormal thermal denaturation stability and the ability to be easily reduced to natural forms after cooling [7]. Some undigested SBM proteins were identified as protease inhibitors in chicken digestive fluids fed SBM diets [8]. Two storage proteins, glycine and β-glycine, were considered to be the main soybean allergens [9,10]. Compared with other plant proteins, they revealed greater resistance to digestive enzymes [8]. Antinutritional factors, such as trypsin inhibitor, glycine and β-conglycinin in SBM, might be the most important factors leading to the failure of broilers to completely digest crude protein and amino acids [11]. About a quarter of the protein in broiler diets was provided by corn, which was mainly composed of gliadin, accounting for more than 60% of all protein in whole grains [12]. The amino acid composition was rich in glutamine and hydrophobic amino acids, which helped the gliadin part of the protein insoluble in water. The relative abundance of zein was highly resistant to protein hydrolysis [13] because the cysteine in grain γ-gliadin was highly conserved [14].

The changes in digestive enzyme levels in GIT affected the digestibility of nutrients to a certain extent. The development of digestive enzyme secretion after hatching may be a limiting factor for digestion and subsequent food intake and growth [15]. Some proteins in SBM and corn had a certain degree of resistance to endogenous proteases, which provided an opportunity to use exogenous proteases in feed to improve protein digestibility.

Protease hydrolysis in vitro has been proven to be an effective method to reduce or eliminate the anti-nutritional factors in SBM, increase the apparent ileal digestibility and improve broiler performance [16]. Additional addition of exogenous proteases could improve amino acid digestibility, feed conversion rate and intestinal integrity of broilers fed normal [17] or low-protein diets [18,19]. In addition, one study demonstrated that the addition of AlP (optimal pH 9.0) increased ileal energy digestibility in 35-day-old broilers [20].

Microbial proteases had the advantages of animal and plant proteases, and could improve the disadvantages of high production cost, low enzyme production efficiency and difficulty achieving large-scale production. Protein-producing microorganisms mainly included bacteria, molds and actinomycetes. Microbial proteases were commonly used in feed to improve animal protein utilization and reduce nitrogen excretion. Based on different optimal pH, proteases can be divided into acidic protease (AcP), alkaline protease (AlP) and neutral protease (NeP). It was reported that the average pH values of the crop, gizzard, proventriculus, proximal and distal intestinal contents were 4.0–7.8, 0.3–4.1, 0.4–5.4, 5.2~7.6 and 5.5~7.7, respectively [21]. The pH of the contents of different digestive organs in broilers is different, and might be too low in the gizzard or proventriculus and too high in the small intestine, which is not conducive to the maximum effect of exogenously added enzymes. Many studies have shown that the enzyme activity in feed is affected by the pH value of the digestive tract. For example, xylanase had high activity at pH 6.0–7.0 and no activity at pH 3.0 [21,22]; β-glucanase had high activity at pH 3.0~7.0 [21,23]; the amylase had high activity at pH 6.0 and 6.5, but no activity or very low activity at pH 3.0, 7.0 and 7.5; α-galactosidase had high activity at pH 6 and no activity at other pH levels. The protease had no or very low activity at all levels except pH 3.0 [21]; and phytases from *Aspergillus oryzae* and *Aspergillus niger* confirmed the best activity at pH 4.0 and nearly pH 5.0, respectively [24]. The pH level in the digestive tract of broilers might be an important limiting factor for the maximum activity of exogenous proteases.

The efficacy of exogenous proteases was influenced by commercial sources of fungi or bacteria [25,26,27,28], optimum pH or temperature characteristics [25,27,28], addition levels [20] and combination levels [18]. Most proteases in published studies were single-component proteases or commercial products, but the effects of different protease combinations on broiler growth and protein digestibility have not been widely reported. The AcP, NeP and AlP selected in this study are serine proteases such as pepsin and chymotrypsin, and have similar action sites, while keratinase (Ker) has the characteristics of hydrolyzing hydrophobic amino acids. The purpose of this study was to investigate the effects of single addition of AcP, NeP, AlP and Ker and their combination on in vitro and in vivo protein digestibility of broilers fed corn–soybean meal diets from the perspective of insufficient secretion of endogenous enzymes in broilers and the effect of exogenous enzymes on endogenous enzymes.

## 2. Materials and Methods

The experiment was conducted in the State Key Laboratory of Animal Nutrition. The experimental protocols used in the current study were reviewed and approved by the Animal Care and Use Committee of Institute of Animal Science, Chinese Academy of Agricultural Sciences (2017-007).

### 2.1. Animals and Proteases

A total of 640 one-day-old Arbor Acres+ (AA+) male broilers were purchased from Beijing Huadu Chicken Co., Ltd. and vaccinated. They were randomly divided into 8 groups of 10 with 8 replicates of 10 birds per replicate cage. The temperature of broilers was increased to 35 °C in the early stage, and then decreased by 2 °C per week until being maintained at 25 °C. The broilers were immunized according to the routine immunization degree, and the chicken house was regularly disinfected and well-ventilated during the broiler feeding process. All broilers were free to feed and drink throughout the experiment.

The proteases used in this research were described as AcP from *Aspergillus niger*, AlP and NeP from *Bacillus subtilis* (Jinan Baisijie Biological Engineering Co., Ltd., Jinan, China) and Ker from *Bacillus licheniformis* (our own laboratory) for the in vitro and in vivo studies.

### 2.2. Experimental Design

Corn–soybean meal-based diets were recommended to meet the nutritional requirements of AA+ 1~21- and 22~31-day-old broilers, either alone or in combination with proteases, as shown in Table 1. The detailed process was as follows. According to the addition of different proteases in the diet, these broilers were divided into the control group, 0.8 U/g NeP, 0.8 U/g AlP, 0.4 U/g Ker, 1.6 U/g AcP alone or together with 0.8 U/g NeP, 0.8 U/g ALP or 0.4 U/g Ker groups, and these proteases of four different microorganisms were evaluated in broilers in vitro and in vivo. Dietary chromium oxide (Cr_2_O_3_) was added as an exogenous indicator to evaluate the digestibility of nutrients. Diet composition and nutritional levels are shown in Table 2.

### 2.3. Enzyme Activity In Different pH Buffers

The concentrations of citrate buffer, sodium phosphate, Tris-HCl and glycine-NaOH were 0.2 mol/L, 0.03 mol/L, 0.05 mol/L and 0.05 mol/L, respectively. The buffers were configured and stored at room temperature. The proteolytic activity of 4 proteases under various pH values (citrate buffer, pH 2–6; sodium phosphate, pH 6–8; Tris-HCl, pH 8–9; glycine-NaOH, pH 10–12) was measured by hydrolyzing casein substrate at 40 °C for 30 min according to the Folin phenol reagent method. One unit of protease activity was defined as the amount of enzyme liberated 1 g of tyrosine at 40 °C under the optimal pH. The activity of the highest enzyme detected in the experiment was defined as 100% to evaluate the activity of the remaining enzymes. All reactions were carried out in triplicate.

### 2.4. The Restorability of Four Proteases Treated by Different pH Buffers Was Measured In Vitro

The stability of 4 proteases at gastro (acidic pH 3.3) and small intestine (neutral pH 6.2) pH conditions of broiler was determined by incubating 300 U of each protease with 2 mL of citrate buffer (pH 3.3) for 50 min, or followed by adjusting pH to 6.2 by NaOH for an additional 86 min at 40 °C without substrate. The residual activity of the protease at two stages was assayed at each optimal pH, respectively. The highest enzyme activity was defined as 100% to evaluate the remaining enzyme activities. All reactions were performed in triplicate.

### 2.5. Effects of Four Proteases on Trypsin and Chymotrypsin Activity Were Detected In Vitro

Subsequently, the effects of 4 proteases on trypsin and chymotrypsin activity were conducted in this experiment. The stock solutions of trypsin (about 230,000 U/mL; Sigma, St. Louis, MO, USA) and chymotrypsin (about 25,000 U/mL; Sigma) were prepared in 0.2 M Tris-HCl buffer (pH 7.8) with 20 mM CaCl_2_ according to the manufacturer’s definition of units of activity. The activities of trypsin and chymotrypsin were determined according to Borda-Molina [29] with some modifications, and the benzoyl-dl-arginine-p-nitroanilide (BAPNA) and N-glutary-l-pH-enylalanine-p-nitroanilide (GPPNA) were used as substrates, incubating with or without 4 proteases at final concentrations of 0, 0.2, 0.4, 0.8 and 1.6 U/mL at pH 7.8 and 40 °C for 60 min.

### 2.6. Effects of Different Doses of Four Proteases on Crude Protein Digestibility of Corn Gluten Meal and SBM In Vitro

Under conditions in vitro, the effects of four different doses of proteases on CP digestibility of SBM and corn gluten meal (CGM) in the whole digestive tract (including crop, stomach and intestines) were studied to determine whether the crop of broilers was the relatively optimal position for the four protein hydrolysis feed proteins under weak acid conditions.

According to the in vitro digestion process of the GIT of 31-day-old broilers described by Bryan et al. [30], the following modifications were made. In the process of in vitro digestion, 1.00 g matrix (CGM, soybean meal or a mixture of corn and soybean meal in 7:3) was ground and screened by 60-mesh, and then placed in 50 mL of centrifuge tubes. The pH value was adjusted to 4.91 with 3.5 mL of 1.1 mol/L of HCl, and 0.5 mL of chloramphenicol (30 μg/mL) was added. Subsequently, each protease was dissolved in 0.25 M sodium acetate buffer with the best pH value to different final concentrations, and the final concentrations of protease in the digestive system were ensured to reach 0, 0.2, 0.4, 0.6 and 0.8 U/g feed. Finally, these centrifuge tubes were incubated in an air-bath agitator at 40 °C and shaken at 120 rpm for 50 min to simulate the process of crop digestion.

During the crop digestion period, 1 g of substrate was weighed, 3.5 mL of phosphate buffer (0.1 M, pH = 4.9) was added, 0.5 mL of chloramphenicol solution was added to inhibit microbial growth and the substrate was digested in an incubator at 40 °C at 120 r/min for 50 min. During the gastric digestion period, the pH was adjusted to 3.3 with 0.1 M hydrochloric acid, digested with pepsin (P7012, ≥2500 U/mg)–salt solution and cultured under the same conditions for 61 min.

Subsequently, the pH value was adjusted to 6.02 by adding 1 mL of NaHCO_3_ with a certain concentration, and 26 mL of sodium acetate buffer (pH 6.20) and trypsin from porcine pancreas (P3292, 4× USP; Sigma-Aldrich, St. Louis, MO, USA) was mixed and cultured for 163 min at the same temperature and shaking speed to replicate the digestive process in the small intestine.

At the end of digestion, 5 mL of 20% sulfosalicylic acid was added to precipitate the digestive juice for 30 min, and then centrifuged at 8000 r/min for 30 min. Then, the supernatant was collected in the new centrifuge tube to analyze the amount of CP. The IVDCP was calculated using the following formula:

IVDCP (%) = (CP_sample_ − (CP_Residue_ − CP_Blank_))/CP_Sample_ × 100

### 2.7. Effects of Four Proteases Alone or in Combination on IVDCP of Corn–Soybean Meal Mixture

Further, the effects of adding 4 kinds of protease alone or in combination on the in vitro CP digestibility of corn–soybean meal mixture (7:3) were investigated. The digestion procedure was completely consistent with that in Section 2.6 in vitro.

### 2.8. Effects of Four Proteases on Crude Protein and AA Digestibility of Broilers Fed Corn–Soybean Meal Diet

An animal trial was carried out at the State Key Laboratory of animal nutrition to explore the effects of the supplementation of 4 proteases individually or in combination on the growth, the CP and AA apparent digestibility in broiler-fed corn–soybean meal diet.

### 2.9. Effects of Four Proteases on Growth Performance of Broilers Fed Corn–Soybean Meal Diet

Broilers were weighed on days 1, 22 and 31 of the experiment, and fasted for up to 12 h before weighing. The body weight of each stage was recorded, the addition and loss of feed and the feed intake of each group were recorded in detail and the average daily feed intake (ADFI), average daily gain (ADG) and feed-to-conversion ratio (FCR) were calculated.

### 2.10. Sampling and Analysis of Ileal Digesta

After the broilers were fed normally for 1 h at the age of 31 days, two broilers were randomly selected from each treatment every 2 h. After intravenous injection of anesthetics under the wings, the abdominal cavity of broilers was opened, and the middle 1/3 segment of ileal chyme was taken. The chyme samples of each replicate group were mixed and stored in −20 °C refrigerator quickly.

The contents of CP, AA and Cr in freeze-drying sub-samples of ileal digesta and diets were analyzed. The CP content was analyzed using the Kjeldahl method, and Cr content was determined according to Williams et al. [31]. Additionally, detection of amino acid content was performed as described in Zhou et al. [32].

The digestibility coefficients (DCs) of CP or AAs for each diet were calculated on a pen basis, according to the following formula:

DC_AA(CP)diet_ (%) = 100 − [(Cr_diet_ ×AA_(CP)digesta_)/(Cr_digesta_ ×AA_(CP)diet_)]

Among them, Cr_diet_ and Cr_digesta_ represented the concentration of Cr (g/kg) in diet and digesta samples, respectively. AA_(CP)diet_ and AA_(CP)chyme_ were the respective concentrations (g/kg) of AA (CP) in diet and chyme samples.

### 2.11. Statistical Analysis

SPSS 19.0 (IBM SPSS, Armonk, NY, USA) was used for statistical analysis. All the results were expressed as mean ± mean square error (SEM). Three independent experiments were conducted for each experiment. The significance of the difference between the two groups was analyzed by a double-tailed Student’s *t*-test unless special attention was paid to paired comparison. More than 2 groups were statistically compared through one-way ANOVA, and multiple comparisons were performed with Tukey–Kramer correction. All statements of significance were considered on a *p*-value less than 0.05 unless otherwise specified.

## 3. Results

### 3.1. The Activity of Four Exogenous Proteases in Different pH Buffers

**Figure 1:**
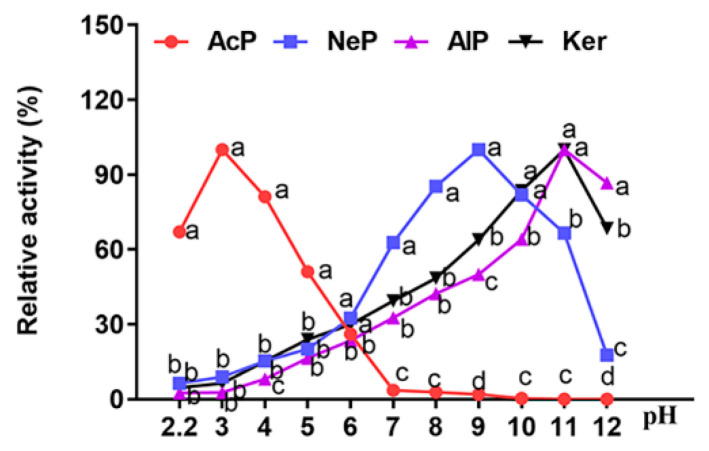
The proteolytic activity of AcP, NeP, Ker and AlP in different pH buffers. ^a^ *p* < 0.05 or ^d^ *p* < 0.05 compared with NeP, AlP or Ker group; ^b^ *p* < 0.05 compared with AcP, NeP or AlP group; ^c^ *p* < 0.05 compared with AcP, NeP or Ker group.

The activity of four proteases hydrolyzing casein substrate under different pHs is shown in Figure 1. The optimal pH values of AcP, NeP, AlP and Ker were 3.0, 9.0, 11.0 and 11.0, respectively. The activities of acidic protease can be maintained at more than 50% in the range of pH 2.2 to 5.0, and little activity was detected as pH was raised to above 7.0.

Less than 50% of NeP activity was reserved between pH 6.0–7.0 close to the jejunum pH of broilers, and only 10% activity at pH 3.0, which was close to gastric pH. AlP and Ker exhibited similar properties to NeP at different pH values, with lower activity at acidic pH (3.0) and neutral pH (6.0–7.0). These results suggested that the crop and gastro were suitable sites for acidic proteases to hydrolyze feed protein, and crop and small intestines for NeP, AlP and Ker.

### 3.2. The pH Values on Recoverability of Four Exogenous Proteases

**Figure 2:**
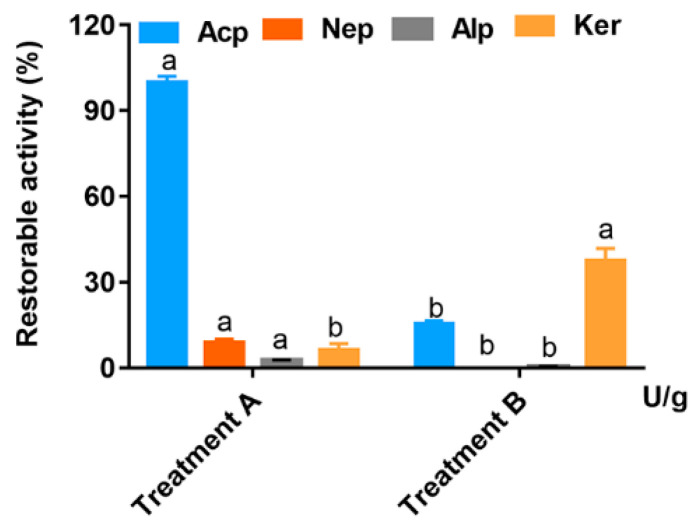
Storability of AcP NeP, AlP and Ker incubated at pH 3.3 for 50 min (treatment A) or from pH 3.3 at 50 min followed by incubation at pH 6.2 for 68 min (treatment B) at 40 °C. Enzyme activity without incubation was considered 100%. Significant differences are shown by bars labeled with various letters. ^a^ *p* < 0.05 compared with AcP, NeP or AlP group in treatment B; ^b^ *p* < 0.05 compared with Ker group in treatment B.

The recoverability of four proteases at pH 3 and 6.2 are shown in Figure 2. In this research, AcP was stable in pH 3.3 buffer at 37 °C for 50 min with little loss of activity, but was sensitive to neutral pH 6.2, retaining only 15% of the protein hydrolyzate from pH 6.2. NeP, AlP and Ker were sensitive to low pH, with negligible residual activity after incubation in acidic pH 3.3 at 37 °C for 50 min. The recoverable activities of AcP, NeP, AlP and Ker at pH 3.3 to 6.2 were 15.31%, 0%, 0.48% and 37.68%, respectively. Additionally, Ker showed better recoverability from pH 3.3 to pH 6.2. It was revealed that the small intestine may be the main site of action of NeP, AlP and Ker.

### 3.3. Four Exogenous Proteases on Trypsin and Chymotrypsin Activities

**Figure 3:**
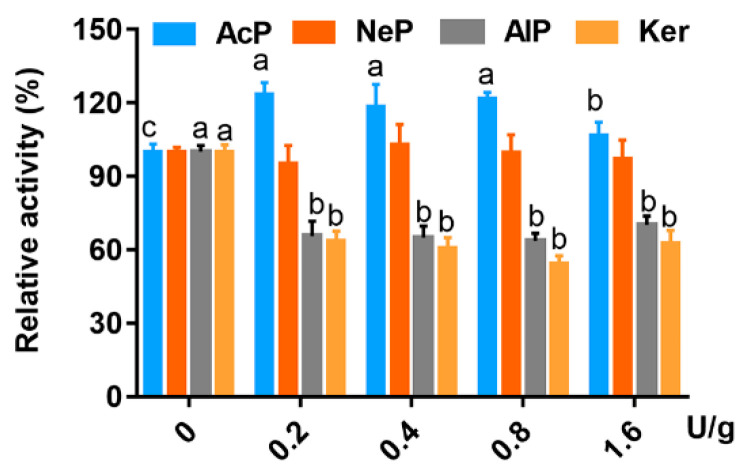
Effects of four proteases incubated with trypsin at different final concentrations (0, 0.2, 0.4, 0.8 and 1.6 U/g) on the relative activity of trypsin (%). Significant differences are shown by bars labeled with various letters. ^a^ *p* < 0.05 compared with AlP or Ker group incubated with 0.2, 0.4, 0.8 or 1.6 U/g of trypsin; ^b^ *p* < 0.05 compared with AlP or Ker group incubated with 0.8 or 1.6 U/g of trypsin; ^c^ *p* < 0.05 compared with AlP, NeP or Ker group incubated with 0.2, 0.4, 0.8 or 1.6 U/g of trypsin.

**Figure 4:**
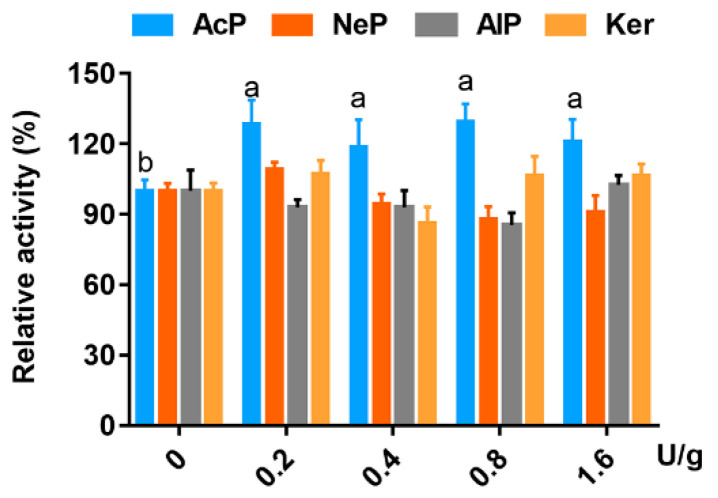
Relative activity of chymotrypsin (%) incubated with or without four proteases in final concentrations of 0, 0.2, 0.4, 0.8 and 1.6 U/g. Significant differences are shown by bars labeled with various letters. ^a^ *p* < 0.05 compared with AcP group incubated with 0 U/g of trypsin; ^b^ *p* < 0.05 compared with AcP group incubated with 0.2, 0.4, 0.8 or 1.6 U/g of trypsin.

The effects of proteases on the activity of trypsin and chymotrypsin are presented in Figure 3 and Figure 4, respectively. The activity of trypsin was improved by all levels of AcP, and increased significantly by 0.2, 0.4, 0.8 and 1.6 U/g at 23.5%,18.7%, 21.8% and 6.8%, respectively, but was decreased by AlP and Ker dramatically. NeP did not affect trypsin activity. The effect of AcP on chymotrypsin activity was similar to trypsin, but NeP, AlP and Ker had little influence on the activity of chymotrypsin.

### 3.4. Effects of Different Doses of Exogenous Proteases on Crude Protein Digestibility of CGM and SBM In Vitro

The effects of four different doses of protease on the in vitro crude protein digestibility (IVCPD) of CGM and SBM are shown in Table 3 and Table 4. In general, the effects of AcP, NeP, AlP and Ker on IVCPD of SBM and CGM were roughly dose-dependent (*p* < 0.05), but the optimal enzyme activity addition of each enzyme was different. Except for Acp and Ker (the optimum enzyme activity was 1.6 U/g), the optimum enzyme activity of the other two enzymes to improve the digestibility of SBM crude protein was 0.8 U/g (Table 3). Compared with the control group, the proportions of IVCPD increased by the optimal amount of Acp, Nep, Alp and Ker were 14.34%, 14.37%, 14.81% and 10.65%, respectively. These results suggested that different sources of enzymes and different doses of enzymes had different effects on the IVCPD of SBM.

The improvement effect of four proteases with different enzyme activities on IVCPD of CGM was different. The optimum enzyme activities of AcP, NeP, AlP and Ker were 0.8 U/g, 1.6 U/g, 0.4 U/g and 0.2 U/g, respectively (Table 4). Compared with the control, AcP, NeP, AlP and Ker increased the relative levels of IVCPD of SBM by 42.24%, 47.31%, 91.38% and 64.83%, respectively. The above results revealed that the improvement effect of four exogenous enzymes on IVCPD of CGM was better than that of SBM, and the improvement effect of Ker and AlP was relatively obvious.

### 3.5. Crude Protein Digestibility of a Mixture of Corn and SBM with or without Four Proteases Alone or in Combination In Vitro

The optimal levels of the four proteases to increase CGM and SBM IVCPD are shown in Table 3 and Table 4. Subsequently, the effects of proteases on corn–SBM (7:3) mixed diets were screened by comprehensively considering the improvement effects of four proteases on the IVCPD of corn and SBM. The individual dosages of AcP, NeP, AlP, and Ker were 1.6, 0.8, 0.8, and 0.4 U/g, respectively, and their combinations were at the same level as AcP together with NeP, AlP or Ker. The effect of the addition of proteases alone or in combination on the IVCPD of a 7:3 ratio corn and SBM mixture is shown in Table 5. Compared with the control, AcP alone or together with NeP, AlP or Ker significantly increased IVCPD (*p* < 0.05), while adding NeP, AlP or Ker alone had no significant effect (*p* > 0.05). Supplementation with AcP alone was more effective for IVCPD than any other treatment. The combination of AcP and NeP, AlP or Ker further increased IVCPD compared to NeP, AlP and Ker provided alone (*p* < 0.05).

### 3.6. Growth Performance and Apparent Ileal AA Digestibility In Vivo

Compared with the control group, the addition of ACP or NeP alone, or ACP combined with NeP or Ker alone, significantly increased the DFI of broilers during the growing period (day 22 to day 31) (*p* < 0.05). However, ACP combined with AlP or Ker significantly reduced the DFI of broilers from day 1 to day 31 (*p* < 0.05); in addition, compared with the control group, the addition of AcP, NeP or AlP alone and the combined addition of AcP and Ker significantly increased the ADG of broilers from day 1 to day 31, while the addition of AcP combined with AlP significantly decreased the ADG of broilers from day 1 to day 31 (*p* < 0.05) (Table 6). The apparent ileal digestibility of CP in a broiler of 31 days was significantly reduced by the addition of four proteases (*p* < 0.05) whatever the supplemented individual or combined form (Table 7). Ker alone or together with AcP showed a better performance than other protease supplementation (*p* < 0.05), but lower than the control group (*p* < 0.05). The addition of Ker alone and AcP combined with NeP supplementation increased the apparent ileal digestibility of Tyr (*p* < 0.05) (Table 8). Adding Ker alone and AcP combined with NeP, AlP or Ker all significantly increased Pro apparent ileal digestibility (*p* < 0.05). Compared with the control group, the apparent ileal digestibility of Asp, Thr, Ser, Glu, Gly, Ala, Cys, Met and Phe were decreased after adding all proteases (*p* < 0.05), including alone and in combination.

## 4. Discussion

Microbial proteases are usually used in feed to improve protein utilization and reduce nitrogen excretion. Proteases were divided into AcP, NeP and AlP according to their optimum pH for activation. The average pH values of crop, stomach, proximal and distal small intestine of broilers were 6.5, 3.0, 7.0 and 7.5, respectively [21]. A previous study in our laboratory demonstrated that the average pH values of crop, stomach and jejunum contents of broilers at 31 days were 5.0, 3.3 and 6.2 (unpublished), respectively. Factors such as breed, growth stage, feed, and growth environment of broiler chickens might cause differences in pH in the contents of different organs. The widespread use of exogenous proteases was limited by the instability of animal stomachs and small intestines at acidic or neutral pH conditions; thus, well low pH adaptability has become one of the most valuable properties of proteases in animal feed.

The activity of the enzyme is affected by pH, and the enzyme usually exerts the greatest effect under optimal pH conditions [33]. To study the effects of different exogenous enzymes on protein utilization in broiler diets, mainly due to the different pH of the digestive parts of broiler chickens [33], considering the characteristics of four kinds of exogenous proteases and the difference in pH environment from crop to the stomach and small intestine during broiler development, the effects of different exogenous proteases on the protein digestibility of broilers were studied. AcP, NeP and AlP efficiently hydrolyze animal and plant proteins under acidic, neutral and alkaline conditions, respectively, and hydrolyze macromolecular proteins into small molecular peptides or amino acids to facilitate the effective absorption and utilization of proteins [34]. Ker is a special alkaline serine protease containing disulfide bond hydrolase and polypeptide hydrolase, which can efficiently open disulfide bonds and degrade keratin, gliadin, polypeptide and other proteins [35]. The location and degradation rate of AcP, NeP, AlP and Ker in broiler chickens are also two factors that affect the function of four proteases. Studies have shown that the main action sites of AcP, NeP and AlP in broilers are the crop, stomach and small intestine, respectively [7,36]. However, the position of Ker in broilers has not been extensively studied. The optimum pH of AcP, NeP, AlP and Ker were 3.0, 9.0, 11.0 and 11.0, respectively. Combined with the previous research results of our laboratory (unpublished), the average pH of crop, gizzard, glandular stomach and jejunum contents of broilers at 31 d was 4.91, 3.37, 3.37 and 6.20, respectively. The activities of AcP, NeP, AlP and Ker may also be inhibited when the pH of the broiler digestive tract changes. Indeed, our in vitro study confirmed that the refolding rate of acid protease was 15.31% and the refolding rate of keratinase was 37.68% when the acid condition (pH 3.3) was adjusted to weak acid (pH 6.2), while the refolding rate of the other two enzymes was negligible. Under the conditions of this study, the selection of proteases with a wide range of acid resistance is helpful for exogenous enzymes to exert their effects in different parts of broilers, thereby improving the enzymatic hydrolysis efficiency of proteins.

The protein digestibility of exogenous proteases on CGM and SBM and the mixture of corn and SBM were the focus of our attention. The results showed that the optimum addition of AcP, NeP, AlP and Ker to improve the crude protein digestibility of soybean meal was 1.6, 0.8, 0.8 and 1.6 U/g, and the proportion of IVCPD increased by SBM was 14.34%, 14.37%, 14.81% and 10.65%, respectively. The optimal enzyme activities of AcP, NeP, AlP and Ker were 0.8, 1.6, 0.4 and 0.2 U/g, respectively, and the proportions of increasing CGM IVCPD were 42.24%, 47.31%, 91.38% and 64.83%, respectively. These results indicate that the effect of four exogenous enzymes on the IVCPD of CGM is better than that of SBM, which may be due to the different protein structures of the two diets. The protein of CGM is mainly gliadin, glutelin, globulin and albumin. The natural corn protein peptide chain is curled into a compact sphere, and the structure is relatively stable. By adding protease to destroy the structure of corn protein, exposing the contact site with the enzyme, and increasing the action point of the enzyme, the enzymatic hydrolysis rate can be improved. In addition, we also found that AlP was superior to the other three exogenous proteases in improving IVCPD of SBM. The enzymes used to hydrolyze SBM can be acid protease, alkaline protease and neutral protease, and alkaline protease is widely used [37,38,39].

From our results, the addition of protease had a greater effect on the DFI of broiler chickens during the brooding period (days 21 to 31), and the effect of AcP and NeP alone or in combination was more significant. It can be seen that AcP and NeP had a synergistic effect on improving the feed intake of broiler chickens during the brooding period. In addition, AcP and Ker had a synergistic effect in improving ADG of broilers, while AcP and AlP had an antagonistic effect. Considering that the optimum pH span between AcP and AlP is large, it is necessary to explore the effect of batch treatment on the ADG of broilers.

There were anti-nutritional factors, such as soybean antigen protein, trypsin inhibitor and plant lectin in soybean meal, which seriously affected the hydrolysis of a soybean meal protein by exogenous protease [40]. Our results showed that the addition of four proteases alone and in combination significantly reduced the apparent ileal digestibility of CP in broilers at 31 days, especially the apparent ileal digestibility of Asp, Thr, Ser, Glu, Gly, Ala, Cys, Met and Phe. This was not consistent with in vitro evaluation results, which might be due to in vitro evaluation of proteases using a single feed ingredient, or animal hormones, such as regulatory effects [41]. Although a large amount of evidence showed that exogenous protease supplementation could make up for the deficiency of endogenous enzymes in broilers and promote the utilization of protein and amino acids [42,43], the effect of protease supplementation was related to the dosages [43]. When the dosage was too high, it might inhibit the secretion of endogenous protease in a feedback manner. Therefore, the addition of high-dose protease in the experiment might not be conducive to improving nutrient digestibility.

The best exogenous enzymes and combinations were screened. In short, the evaluation and screening of protease is still a more complex problem, not only need to consider the digestibility of crude protein, but also need to consider the digestibility of amino acids, nitrogen deposition and metabolism in order to accurately reflect the true effect of protease, protease to achieve scientific evaluation.

## 5. Conclusions

The application of proteases in broiler diets is complex. Exogenous proteases were not only sensitive to intestinal conditions (pH and endogenous protease), but also harmed trypsin and chymotrypsin activities in in vitro studies. In addition, exogenous proteases could improve the growth performance of broilers, but decrease the ileal digestibility of crude protein in vivo. Therefore, the characteristics and dosage of protease and the protein level in feed should be comprehensively considered when providing protease in animal feed.

## Figures and Tables

**Table 1:** The experiment design and treatments.

Treatments	Levels (U/g Diet)
Control	0
AcP	1.6
NeP	0.8
AlP	0.8
Ker	0.4
AcP + NeP	1.6 + 0.8
AcP + AlP	1.6 + 0.8
AcP + Ker	1.6 + 0.4

AcP, acidic protease; NeP, neutral protease; AlP, alkaline protease; Ker, keratinase.

**Table 2:** Composition and nutrient profile of the basal diet (%).

Ingredients	Days 0–21	Days 22–31
Corn	58.75	61.45
Soybean meal 44% CP	34.50	31.00
Soya oil	2.70	3.80
CaHPO_4_	1.90	1.60
Limestone	1.20	1.30
NaCl	0.35	0.30
Micro-mineral Premix ^a^	0.20	0.20
Vitamin Premix ^b^	0.02	0.02
DL-Met	0.18	0.10
L-Lys	0.1	0.15
Choline chloride	0.10	0.08
Total	100.00	100.00
Calculated nutritive value		
Metabolizable energy (kcal/kg)	3040.00	3100.00
Crude protein	20.46	19.76
Calcium	0.98	0.90
Available phosphorus	0.45	0.40
Lysine	1.12	1.11
Methionine	0.52	0.40
Threonine	0.78	0.78

^a.^ The premix provided the following per kg of diets: Cu, 8 mg; Fe, 80 mg; Zn, 75 mg; Mn, 100 mg; Se, 0.30 mg; I, 0.35 mg. ^b.^ The multi-vitamin provided the following per kg of diets: vitamin A, 10,000 IU; vitamin D3, 2600 IU; vitamin B1, 2 mg; vitamin B2, 3. 6 mg; pantothenic acid, 12 mg; vitamin B6, 3 mg; vitamin B12, 0.024 mg; vitamin E, 20 IU; vitamin K3, 2 mg; nicotinic acid, 10 mg; folic acid, 0.8 mg; biotin, 0.12 mg.

**Table 3:** The IVCPD (%) of SBM with or without four proteases in different doses.

Protease Dosage (U/g)	AcP	NeP	AlP	Ker
0	57.66 ^c^	57.66 ^c^	57.66 ^c^	57.66 ^c^
0.2	60.13 ^b^	62.01 ^b^	63.11 ^b^	58.58 ^c^
0.4	61.05 ^b^	62.16 ^b^	62.50 ^b^	61.85 ^b^
0.8	60.62 ^b^	65.95 ^a^	66.20 ^a^	62.45 ^b^
1.6	65.93 ^a^	63.38 ^b^	64.28 ^ab^	63.80 ^a^
SEM	0.283	0.652	1.138	0.620
*p*-value	<0.001	<0.001	<0.001	<0.001

Means with different superscript letters differ (*p* < 0.05). AcP, acidic protease; NeP, neutral protease; AlP, alkaline protease; Ker, keratinase. ^a^ *p* < 0.05 compared with 0, 0.2, 0.4 or 0.8 U/g of AcP or Ker group, 0, 0.2, 0.4 or 1.6 U/g of NeP group or 0, 0.2 or 0.4 U/g of AlP group; ^b^ *p* < 0.05 compared with 0 or 1.6 U/g of AcP, 0 or 0.8 U/g of NeP or AlP group or 0, 0.2 or 1.6 U/g of Ker group; ^c^ *p* < 0.05 compared with 0.2, 0.4, 0.8 or 1.6 U/g of AcP, NeP or AlP group or 0.4, 0.8 or 1.6 U/g of Ker group.

**Table 4:** The IVCPD (%) of CGM with or without four proteases in different doses.

Protease Dosage (U/g)	AcP	NeP	AlP	Ker
0	21.92 ^c^	21.92 ^d^	21.92 ^d^	21.92 ^e^
0.2	22.32 ^c^	22.52 ^d^	39.05 ^b^	36.13 ^a^
0.4	21.96 ^c^	23.76 ^c^	42.39 ^a^	31.43 ^b^
0.8	31.18 ^a^	30.13 ^b^	39.40 ^b^	28.92 ^c^
1.6	28.90 ^b^	32.29 ^a^	38.61 ^b^	24.24 ^d^
SEM	0.423	0.401	0.727	4.761
*p*-value	<0.001	<0.001	<0.001	0.008

Means with different superscript letters differ (*p* < 0.05). AcP, acidic protease; NeP, neutral protease; AlP, alkaline protease; Ker, keratinase. ^a^ *p* < 0.05 compared with 0, 0.2, 0.4 or 1.6 U/g of AcP; 0, 0.2, 0.4 or 0.8 U/g of NeP group, 0, 0.2, 0.8 or 1.6 U/g of AlP group, or 0, 0.4, 0.8 or 1.6 U/g of Ker group; ^b^ *p* < 0.05 compared with 0, 0.2, 0.4 or 0.8 U/g of AcP, 0, 0.2, 0.4 or 1.6 U/g of NeP, 0 or 0.4 U/g of AlP group, or 0, 0.2, 0.8 or 1.6 U/g of Ker group; ^c^ *p* < 0.05 compared with 0.8 or 1.6 U/g of AcP, 0, 0.2, 0.8 or 1.6 U/g of NeP or 0, 0.2, 0.4 or 1.6 U/g of Ker group; ^d^ *p* < 0.05 compared with 0.4, 0.8 or 1.6 U/g of NeP, 0.2, 0.4, 0.8 or 1.6 U/g of AlP, 0, 0.2, 0.4 or 1.6 U/g of Ker group. ^e^ *p* < 0.05 compared with 0.2, 0.4, 0.8 or 1.6 U/g of Ker group.

**Table 5:** The IVCPD of corn and SBM mixture with or without four proteases individually or in combination.

Treatments	Protease Dosage (U/g)	Digestibility (%)
Control	0	77.49 ^c^
AcP	1.6	86.82 ^a^
NeP	0.8	79.33 ^bc^
AlP	0.8	80.21 ^bc^
Ker	0.4	77.71 ^c^
AcP + NeP	1.6 + 0.8	86.36 ^a^
AcP + AlP	1.6 + 0.8	85.68 ^a^
AcP + Ker	1.6 + 0.4	80.63 ^b^
SEM	-	0.778
*p*-value	-	<0.001

Means with different superscript letters differ (*p* < 0.05). AcP, acidic protease; NeP, neutral protease; AlP, alkaline protease; Ker, keratinase. ^a^ *p* < 0.05 compared with control, AcP, NeP, AlP, AcP together with Ker group; ^b^ *p* < 0.05 compared with control, AcP, Ker, AcP together with AlP or Ker group; ^c^ *p* < 0.05 compared with AcP alone or together with NeP, AlP or Ker group.

**Table 6:** Growth performance of broilers (1 day to 31 days) with or without four proteases individually or in combination.

Treatments	Days 1–21			Days 22–31			Days 1–31		
	ADG (g)	DFI(g)	FCR	ADG (g)	DFI(g)	FCR	ADG (g)	DFI(g)	FCR
Control	50.52	75.92	1.51	70.92	107.91 ^de^	1.52	68.96 ^e^	101.25 ^a^	1.47
AcP	51.50	76.95	1.49	75.06	115.08 ^a^	1.53	70.94 ^a^	104.18 ^a^	1.47
NeP	50.29	76.87	1.53	71.60	110.69 ^bc^	1.54	69.41 ^d^	102.68 ^a^	1.48
AlP	50.71	74.86	1.47	71.23	110.23 ^bcd^	1.54	69.57 ^c^	101.27 ^a^	1.46
Ker	49.60	73.69	1.49	69.59	106.77 ^e^	1.53	68.91 ^e^	99.81 ^a^	1.46
AcP + NeP	49.87	75.08	1.50	72.29	113.54 ^ab^	1.57	69.01 ^e^	99.80 ^a^	1.45
AcP + AlP	49.59	74.2	1.49	68.12	109 ^cde^	1.60	67.49 ^e^	89.79 ^b^	1.33
AcP + Ker	51.48	77.44	1.50	73.05	111.3 ^bc^	1.52	70.63 ^b^	93.28 ^b^	1.32
SEM	1.172	2.253	0.074	2.788	1.474	0.072	0.05	2.066	0.147
*p*-value	0.573	0.640	0.997	0.384	<0.001	0.946	<0.001	<0.001	0.889

Means with different superscript letters differ (*p* < 0.05). AcP, acidic protease; NeP, neutral protease; AlP, alkaline protease; Ker, keratinase; ADG, average daily gain; DFI, daily feed intake; FCR, feed-to-conversion ratio. ^a^ *p* < 0.05 compared with DFI in control, NeP, AlP, Ker, AcP together with AlP or Ker group from days 22 to 31, ADG in all groups except AcP group or DFI in AcP together with AlP or Ker group from days 1 to 31; ^b^ *p* < 0.05 compared with DFI in control, AcP, Ker, AcP together with AlP group from days 22 to 31, ADG and DFI in all groups from days 1 to 31; ^c^ *p* < 0.05 compared with DFI in control, AcP, Ker, AcP together with NeP from days 22 to 31, ADG in all group from days 1 to 31; ^d^ *p* < 0.05 compared with DFI in AcP, NeP, Ker, AcP together with NeP or Ker group from days 22 to 31, ADG in all group from days 1 to 31; ^e^ *p* < 0.05 compared with AcP, NeP, AlP, AcP together with NeP or Ker group from days 22 to 31, ADG in AcP alone, NeP alone, AlP alone or together with Ker.

**Table 7:** The crude protein apparent ileal digestibility in broilers fed corn soybean meal diet with or without four proteases individually or combination.

Treatments	Digestibility (%)
Control	79.14 ^a^
AcP	73.10 ^c^
NeP	73.55 ^c^
AlP	72.80 ^c^
Ker	76.21 ^b^
AcP + NeP	73.28 ^c^
AcP + AlP	73.90 ^c^
AcP + Ker	76.90 ^b^
SEM	1.828
*p*-value	0.027

Means with different superscript letters differ (*p* < 0.05). AcP, acidic protease; NeP, neutral protease; AlP, alkaline protease; Ker, keratinase. ^a^ *p* < 0.05 compared with NeP, AlP, Ker, AcP alone or together with NeP, AlP or Ker group; ^b^ *p* < 0.05 compared with control, AcP, NeP, AlP, AcP alone or together with NeP; ^c^ *p* < 0.05 compared with control, Ker, AcP together with Ker.

**Table 8:** The AAs apparent ileal digestibility of corn soybean meal diet with monocomponent or combination protease (%).

Items	Control	AcP	NeP	AlP	Ker	AcP + NeP	AcP + AlP	AcP + Ker	SEM	*p*-Value
Asp	77.98 ^a^	68.70 ^d^	71.55 ^c^	72.08 ^c^	75.58 ^b^	70.63 ^cd^	68.70 ^d^	72.4 ^c^	2.324	0.012
Thr	73.02 ^a^	59.41 ^d^	64.72 ^c^	64.45 ^c^	69.08 ^b^	63.71 ^c^	60.04 ^d^	63.39 ^c^	3.185	0.011
Ser	78.09 ^a^	68.27 ^cd^	70.40 ^c^	69.71 ^c^	74.26 ^b^	71.57 ^bc^	65.61 ^d^	70.03 ^c^	3.126	0.035
Glu	86.22 ^a^	79.92 ^cd^	81.57 ^c^	81.53 ^c^	84.29 ^b^	81.65 ^c^	78.94 ^d^	81.43 ^c^	1.777	0.020
Gly	75.16 ^a^	65.22 ^bc^	65.85 ^c^	63.62 ^bc^	68.69 ^cd^	62.11 ^cd^	59.58 ^d^	63.29 ^cd^	3.755	0.025
Ala	75.16 ^a^	65.22 ^bc^	65.85 ^bc^	63.62 ^cd^	68.26 ^b^	62.11 ^cd^	59.58 ^d^	63.29 ^cd^	3.748	0.026
Cys	61.44 ^a^	49.36 ^d^	51.32 ^cd^	50.60 ^cd^	54.13 ^bc^	49.07 ^d^	49.39 ^d^	55.29 ^b^	3.663	0.048
Val	73.58 ^a^	63.50 ^c^	67.73 ^bc^	65.71 ^bc^	69.79 ^ab^	67.07 ^bc^	58.87 ^d^	57.23 ^d^	4.104	0.018
Met	96.89 ^a^	95.05 ^ef^	95.95 ^bc^	95.83 ^cd^	96.33 ^de^	95.45 ^ef^	95.46 ^f^	94.60 ^b^	0.613	0.043
Ile	78.55 ^ab^	70.48 ^d^	75.75 ^bc^	75.82 ^bc^	80.06 ^a^	76.65 ^bc^	74.54 ^c^	69.88 ^d^	2.989	0.040
Leu	82.78 ^a^	74.62 ^c^	78.49 ^b^	78.49 ^b^	82.80 ^a^	80.91 ^a^	77.70 ^b^	78.51 ^b^	2.009	0.013
Tyr	68.78 ^bc^	59.37 ^d^	65.38 ^c^	68.93 ^bc^	76.23 ^a^	74.59 ^a^	67.32 ^bc^	68.80 ^b^	3.292	0.004
Phe	80.22 ^a^	70.69 ^c^	74.43 ^c^	74.08 ^c^	78.48 ^b^	76.35 ^c^	73.63 ^c^	74.99 ^b^	2.139	0.012
Lys	83.14 ^a^	75.61 ^c^	78.36 ^b^	79.44 ^b^	82.76 ^a^	79.73 ^b^	77.86 ^b^	79.70 ^b^	2.057	0.036
His	81.17 ^a^	72.43 ^c^	75.45 ^b^	74.70 ^bc^	79.03 ^a^	75.52 ^b^	72.83 ^bc^	75.41 ^b^	2.559	0.050
Arg	88.42 ^a^	81.27 ^c^	84.21 ^b^	84.14 ^b^	86.95 ^a^	83.62 ^b^	81.86 ^c^	83.90 ^b^	1.657	0.009
Pro	72.97 ^b^	53.45 ^d^	67.24 ^c^	70.25 ^bc^	77.86 ^a^	79.61 ^a^	78.17 ^a^	79.77 ^a^	3.075	<0.001

Means with different superscript letters differ (*p* < 0.05). AcP, acidic protease; NeP, neutral protease; AlP, alkaline protease; Ker, keratinase; Asp, Asparticacid; Thr, Threonine; Ser, Serine; Glu, Glutamicacid; Gly, Glycine; Ala, Alanine; Cys, Cysteine; Val, Valine; Met, Methionine; Ile, Isoleucine; Leu, Leucine; Tyr, Tyrosine; Phe, Phenylalanine; Lys, Lysine; His, Histidine; Arg, Arginine; Pro, Proline. ^a^ *p* < 0.05 compared with all AAs apparent ileal digestibility in NeP, AlP, Ker, AcP alone or together with NeP, AlP or Ker group; ^b^ *p* < 0.05 or ^c^ *p* < 0.05 compared with all AAs apparent ileal digestibility in control, NeP, AlP, Ker, AcP alone or together with NeP, AlP or Ker group; ^d^ *p* < 0.05 compared with Asp, Thr, Ser, Glu, Gly, Ala, Cys, Val, Met, Ile, Tyr or Pro apparent ileal digestibility in control, AlP, Ker, AcP alone or together with NeP, AlP or Ker group; ^e^ *p* < 0.05 or ^f^ *p* < 0.05 compared with Met apparent ileal digestibility in control, NeP, AlP, AcP together with AlP or Ker group.

## Data Availability

The data used to support the findings of this study are available from the corresponding author upon request.

## References

[1] Beski S.S., Swick R.A., Iji P.A. (2015). Specialized protein products in broiler chicken nutrition: A review. Anim. Nutr..

[2] Huang Q., Wen C., Yan W., Sun C., Gu S., Zheng J., Yang N. (2022). Comparative analysis of the characteristics of digestive organs in broiler chickens with different feed efficiencies. Poult. Sci..

[3] Bryan D., Abbott D.A., Van Kessel A.G., Classen H.L. (2019). In vivo digestion characteristics of protein sources fed to broilers. Poult. Sci..

[4] Zhao D., Liu X. (2023). Purification, identification and evaluation of antioxidant peptides from pea protein hydrolysates. Molecules.

[5] Hu R., Chen G., Li Y. (2020). Production and characterization of antioxidative hydrolysates and peptides from corn gluten meal using papain, ficin, and bromelain. Molecules.

[6] Ibáñez M.A., De Blas C., Cámara L., Mateos G.G. (2020). Chemical composition, protein quality and nutritive value of commercial soybean meals produced from beans from different countries: A meta-analytical study. Anim. Feed. Sci. Technol..

[7] Roychaudhuri R., Sarath G., Zeece M., Markwell J. (2003). Reversible denaturation of the soybean Kunitz trypsin inhibitor. Arch. Biochem. Biophys..

[8] Recoules E., Sabboh-Jourdan H., Narcy A., Lessire M., Harichaux G., Labas V., Duclos M.J., Réhault-Godbert S. (2017). Exploring the in vivo digestion of plant proteins in broiler chickens. Poult. Sci..

[9] Holzhauser T., Wackermann O., Ballmer-Weber B.K., Bindslev-Jensen C., Scibilia J., Perono-Garoffo L., Utsumi S., Poulsen L.K., Vieths S. (2009). Soybean (Glycine max) allergy in Europe: Gly m 5 (beta-conglycinin) and Gly m 6 (glycinin) are potential diagnostic markers for severe allergic reactions to soy. J. Allergy Clin. Immunol..

[10] Amnuaycheewa P., de Mejia E.G. (2010). Purification, characterisation, and quantification of the soy allergen profilin (Gly m 3) in soy products. Food Chem..

[11] Huang K.H., Li X., Ravindran V., Bryden W.L. (2006). Comparison of apparent ileal amino acid digestibility of feed ingredients measured with broilers, layers, and roosters. Poult. Sci..

[12] Hamaker B.R., Mohamed A.A., Habben J.E., Huang C.P., Larkins B.A. (1995). Efficient procedure for extracting maize and sorghum kernel proteins reveals higher prolamin contents than the conventional method. Cereal Chem..

[13] Cabrera-Chávez F., Iametti S., Miriani M., de la Barca A.M., Mamone G., Bonomi F. (2012). Maize prolamins resistant to peptic-tryptic digestion maintain immune-recognition by IgA from some celiac disease patients. Plant Foods Hum. Nutr..

[14] Lee S.H., Hamaker B.R. (2006). Cys155 of 27 kDa maize gamma-zein is a key amino acid to improve its in vitro digestibility. FEBS Lett..

[15] Noy Y., Sklan D. (1995). Digestion and absorption in the young chick. Poult. Sci..

[16] Angel C.R., Saylor W., Vieira S.L., Ward N. (2011). Effects of a monocomponent protease on performance and protein utilization in 7- to 22-day-old broiler chickens. Poult. Sci..

[17] Erdaw M.M., Perez-Maldonado R.A., Iji P.A. (2018). Supplementation of broiler diets with high levels of microbial protease and phytase enables partial replacement of commercial soybean meal with raw, full-fat soybean. J. Anim. Physiol. Anim. Nutr..

[18] Law F.L., Zulkifli I., Soleimani A.F., Liang J.B., Awad E.A. (2018). The effects of low-protein diets and protease supplementation on broiler chickens in a hot and humid tropical environment. Asian-Australas J. Anim. Sci..

[19] Ndazigaruye G., Kim D.H., Kang C.W., Kang K.R., Joo Y.J., Lee S.R., Lee K.W. (2019). Effects of low-protein diets and exogenous protease on growth performance, carcass traits, intestinal morphology, cecal volatile fatty acids and serum parameters in Broilers. Animals.

[20] Fru-Nji F., Kluenter A.M., Fischer M., Pontoppidan K. (2011). A feed serine protease improves broiler performance and increases protein and energy digestibility. Poult. Sci..

[21] Ao T., Cantor A.H., Pescatore A.J., Pierce J.L. (2008). In vitro evaluation of feed-grade enzyme activity at pH levels simulating various parts of the avian digestive tract. Anim. Feed. Sci. Technol..

[22] Thacker P.A., Baas T.C. (1996). Effects of gastric pH on the activity of exogenous pentosanase and the effect of pentosanase supplementation of the diet on the performance of growing-finishing pigs. Anim Feed. Sci Technol..

[23] Baas T.C., Thacker P.A. (1996). Impact of gastric pH on dietary enzyme activity and survivability in swine fed β-glucanase supplemented diets. Can. J. Anim. Sci..

[24] Naves L.P., Corrêa A.D., Bertechini A.G., Gomide E.M., Santos C.D. (2012). Effect of ph and temperature on the activity of phytase products used in broiler nutrition. Braz. J. Poult. Sci..

[25] Ghazi S., Rooke J.A., Galbraith H., Bedford M.R. (2002). The potential for the improvement of the nutritive value of soya-bean meal by different proteases in broiler chicks and broiler cockerels. Br. Poult. Sci..

[26] Wang H., Guo Y., Shih J.C.H. (2008). Effects of dietary supplementation of keratinase on growth performance, nitrogen retention and intestinal morphology of broiler chickens fed diets with soybean and cottonseed meals. Anim. Feed. Sci. Technol..

[27] Mahmood T., Mirza M.A., Nawaz H., Shahid M. (2017). Effect of different exogenous proteases on growth performance, nutrient digestibility, and carcass response in broiler chickens fed poultry by-product meal-based diets for 35 d. Livest. Sci..

[28] Zendzian E.N., Barnard E.A. (1967). Distributions of pancreatic ribonuclease, chymotrypsin, and trypsin in vertebrates. Arch. Biochem. Biophys..

[29] Borda-Molina D., Zuber T., Siegert W., Camarinha-Silva A., Rodehutscord M. (2019). Effects of protease and phytase supplements on small intestinal microbiota and amino acid digestibility in broiler chickens. Poult. Sci..

[30] Bryan D.D.S.L., Abbott D.A., Classen H.L. (2018). Development of an in vitro protein digestibility assay mimicking the chicken digestive tract. Anim. Nutr..

[31] Williams C.H., David D.J., Iismaa O. (1962). The determination of chromic oxide in faeces samples by atomic absorption spectrophotometry. J. Agric. Sci..

[32] Dai Z., Wu Z., Jia S., Wu G. (2014). Analysis of amino acid composition in proteins of animal tissues and foods as pre-column o-phthaldialdehyde derivatives by HPLC with fluorescence detection. J. Chromatogr. B Analyt. Technol. Biomed. Life Sci..

[33] Eed J. (2012). Factors affecting enzyme activity. Essai.

[34] Solanki P., Putatunda C., Kumar A., Bhatia R., Walia A. (2021). Microbial proteases: Ubiquitous enzymes with innumerable uses. 3 Biotech.

[35] Suh H.J., Lee H.K. (2001). Characterization of a keratinolytic serine protease from Bacillus subtilis KS-1. J. Protein Chem..

[36] Ojha B., Singh P.K., Shrivastava N. (2019). Enzymes in the animal feed industry. Enzym. Food Biotechnol..

[37] Singh U., Kaur D., Mishra V., Krishania M. (2022). Combinatorial approach to prepare antioxidative protein hydrolysate from corn gluten meal with dairy whey: Preparation, kinetics, nutritional study and cost analysis. LWT.

[38] Jiang X., Liu X., Xu H., Sun Y., Zhang Y., Wang Y. (2021). Improvement of the nutritional, antioxidant and bioavailability properties of corn gluten-wheat bran mixture fermented with lactic acid bacteria and acid protease. LWT.

[39] Li H.M., Hu X., Guo P., Fu P., Xu L., Zhang X.Z. (2010). Antioxidant properties and possible mode of action of corn protein peptides and zein peptides. J. Food Biochem..

[40] Mukherjee R., Chakraborty R., Dutta A. (2016). Role of fermentation in improving nutritional quality of soybean meal-a review. Asian-Austral J. Anim..

[41] Cowieson A.J., Roos F.F. (2013). Bioefficacy of a mono-component protease in the diets of pigs and poultry: A meta-analysis of effect on ileal amino acid digestibility. J. Appl. Anim. Nutr..

[42] Cowieson A.J., Abdollahi M.R., Zaefarian F., Pappenberger G., Ravindran V. (2018). The effect of a mono-component exogenous protease and graded concentrations of ascorbic acid on the performance, nutrient digestibility and intestinal architecture of broiler chickens. Anim. Feed. Sci. Technol..

[43] Kaczmarek S.A., Rogiewicz A., Mogielnicka M., Rutkowski A., Jones R.O., Slominski B.A. (2014). The effect of protease, amylase, and nonstarch polysaccharide-degrading enzyme supplementation on nutrient utilization and growth performance of broiler chickens fed corn-soybean meal-based diets. Poult. Sci..

